# A new high-pressure form of Mg_2_SiO_4_ highlighting diffusionless phase transitions of olivine

**DOI:** 10.1038/s41598-017-17698-z

**Published:** 2017-12-11

**Authors:** Naotaka Tomioka, Takuo Okuchi

**Affiliations:** 1 Kochi Institute for Core Sample Research, Japan Agency for Marine-Earth Science and Technology, Kochi, 783-8502 Japan; 2 Hiroshima Institute of Plate Convergence Region Research, Hiroshima University, Hiroshima, 739-8526 Japan; 3 Institute for Planetary Materials, Okayama University, Tottori, 682-0193 Japan

## Abstract

High-pressure polymorphism of olivine (α-phase of Mg_2_SiO_4_) is of particular interest for geophysicists aiming to understand the structure and dynamics of the Earth’s interior because of olivine’s prominent abundance in the upper mantle. Therefore, natural and synthetic olivine polymorphs have been actively studied in the past half century. Here, we report a new high-pressure polymorph, the ε*-phase, which was discovered in a heavily shocked meteorite. It occurs as nanoscale lamellae and has a topotaxial relationship with the host ringwoodite (γ-phase of Mg_2_SiO_4_). Olivine in the host rock entrapped in a shock-induced melt vein initially transformed into polycrystalline ringwoodite through a nucleation and growth mechanism. The ringwoodite grains then coherently converted into the ε*-phase by shear transformation during subsequent pressure release. This intermediate metastable phase can be formed by all Mg_2_SiO_4_ polymorphs via a shear transformation mechanism. Here, we propose high-pressure transformations of olivine that are enhanced by diffusionless processes, not only in shocked meteorites but also in thick and cold lithosphere subducting into the deep Earth.

## Introduction

Phase equilibrium studies confirmed that olivine successively transforms into wadsleyite (β-phase with a spineloid structure) and ringwoodite (γ-phase with a spinel structure) with increasing pressure^1^ (Supplementary Fig. S1). Both polymorphs are considered to be major constituents of the mantle transition zone (MTZ: 410–660 km). At ~23 GPa, corresponding to the uppermost lower mantle (660 km), ringwoodite breaks down to MgSiO_3_-perovskite (bridgmanite) and rock salt-type MgO (periclase)^2^. Only a few natural examples of such high-pressure phases of olivine were discovered in the deep Earth^3,4^. However, during the past half-century, all these high-pressure phases have been frequently found in many meteorites that experienced high-pressure and high-temperature impact events.

The phase transformation mechanisms of olivine at high pressure and temperature have been enthusiastically studied to elucidate the fate of the lithosphere subducting into the deep mantle^5–10^. Importantly, these transformation mechanisms largely control the kinetics of olivine phase transformations^11–14^. Olivine is metastably preserved in thick and cold lithospheric slabs descending toward the MTZ due to kinetically hindered high-pressure transformations at low temperature^14,15^. Metastable olivine eventually transforms upon pressure and temperature increase after subsequent deep subduction. High-pressure transformations of mantle minerals in general mainly occur by a nucleation and growth mechanism, mostly at grain boundaries. This mechanism is rate-controlled by atomic diffusion and the product phases occur incoherently with respect to the lattices of their parental phases. A lattice-coherent shear mechanism, promoted by coherent shear of oxygen sublattices associated with cation shuffling in interstices, was also proposed to affect the olivine–ringwoodite phase transformation based on a topological study^16^ and was assessed using first-principles energy calculations^17^. This diffusionless mechanism was further experimentally confirmed by transmission electron microscopy (TEM) of a recovered sample of Mg_2_SiO_4_ olivine, which was compressed in a laser-heated diamond anvil cell^5^. In contrast to the olivine–ringwoodite transformation, a shear mechanism is not widely considered to play a role in the olivine–wadsleyite phase transformation due to limited experimental evidence^7,8^.

Planetary scientists have been independently investigating natural occurrences of the high-pressure polymorphs of olivine beyond Earth. They focused onto meteorites because meteorites frequently contain high-pressure minerals that were produced by shock metamorphism in their parent bodies: asteroids, Mars, and Moon^18^. Natural ringwoodite and wadsleyite were first discovered in ordinary chondrites that experienced such shock events^19,20^. Their defect structures were repeatedly characterized by TEM. Based on the occurrence of stacking faults in olivine polymorphs, a shear mechanism is proposed to promote the olivine–spinel transformation^21^. The transformation model for Mg_2_SiO_4_ also predicted the possible occurrence of an intermediate phase, named ‘ε*-phase’, exhibiting the smallest unit cell among all spinel and spineloid structures^21^. However, this phase has neither been discovered in high-pressure syntheses products nor in natural meteorite samples. Based on analytical TEM, we here report the first evidence for the natural occurrence of the ε*-phase in a shocked chondrite and discuss its potential geophysical importance.

## Results and Discussion

### Occurrence of the ε*-phase in a shocked chondrite

We examined the Tenham meteorite that fell in Australia in 1879^22^. The Tenham meteorite is an ordinary L6 chondrite that mainly consists of olivine, low-Ca pyroxene, high-Ca pyroxene, plagioclase, kamacite, and troilite. It is highly shocked [highest shock stage S6 of a scheme of six stages (S1–S6) based on the shock classification^23^] and comprises shock-induced melt veins (shock veins, <1 mm thick; Supplementary Figs S2, S3). This meteorite is one of the renowned samples containing various types of high-pressure silicate minerals in and in the vicinity of shock veins^18^. Based on the high-pressure mineral assemblages reported in the Tenham meteorite and experimental phase equilibrium studies, the peak shock pressure was estimated to be ~25 GPa^24^.

**Figure 1:**
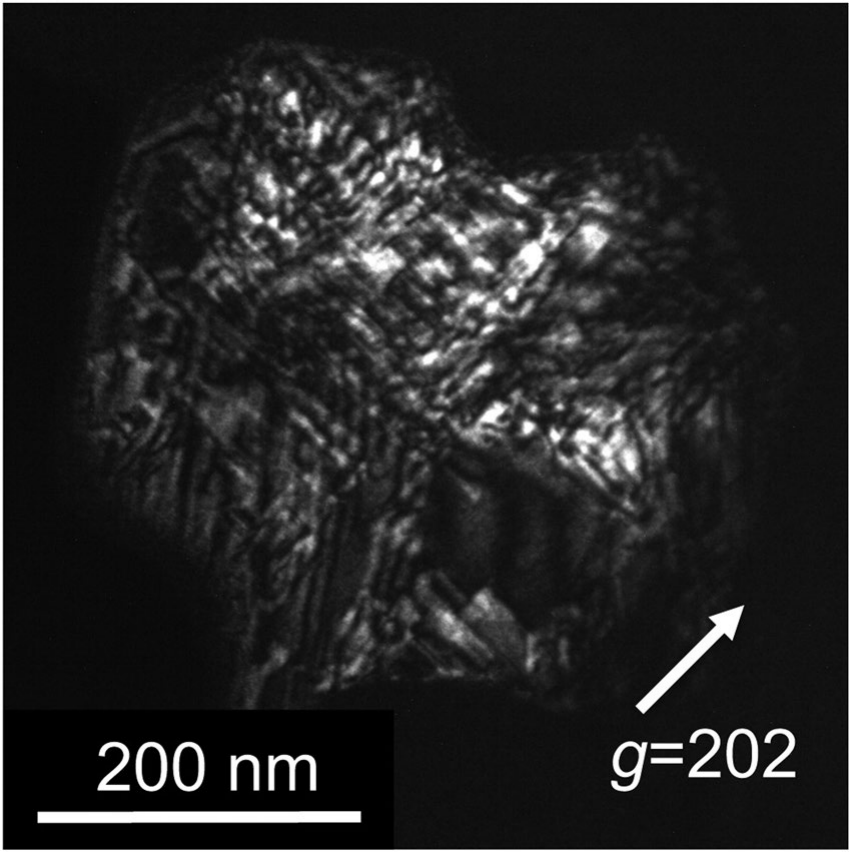
Dark-field (DF) transmission electron micrograph of a ringwoodite grain with high density of stacking faults on {110} planes in the Tenham meteorite. The DF image is formed with a 202 reflection.

**Figure 2:**
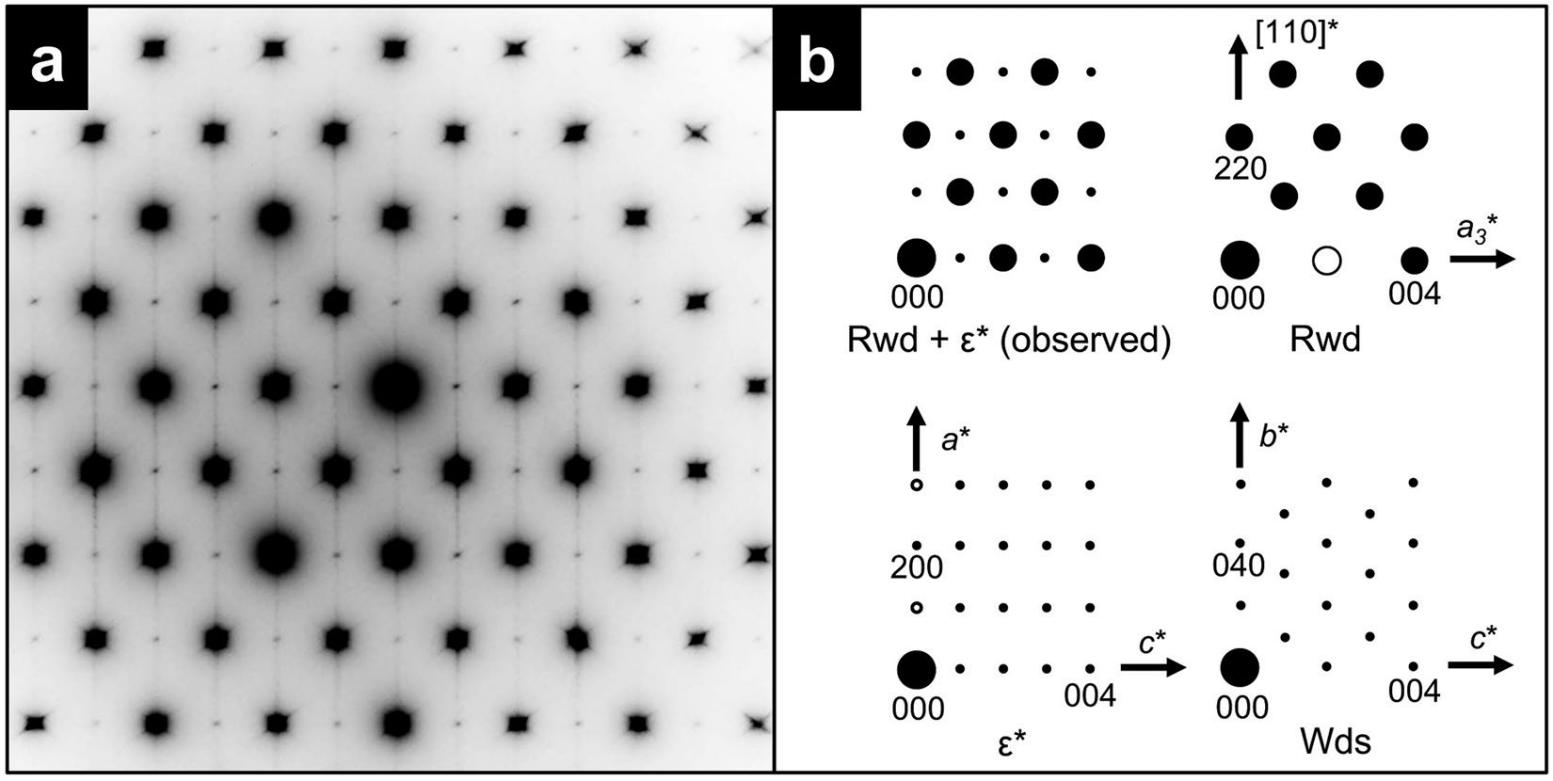
Selected area electron diffraction (SAED) patterns of ringwoodite (Rwd) with the new spineloid ε*-phase. (**a**) SAED pattern along the $[1\,\overline{1}\,0]$ zone axis of the host ringwoodite and ε*-phase therein. (**b**) Schematic illustration of a diffraction pattern in (A) (upper left). The major reflections correspond to ringwoodite (upper right), while weak reflections are due to the ε*-phase (lower left). The filled circles represent diffraction spots satisfying the reflection conditions for the space group symmetries of each phase. The open circles denote diffraction spots that are forbidden but appear due to dynamical diffraction. Streaking along the vertical direction is due to stacking disorder on (110)_Rwd_. The pattern in the lower right is the SAED pattern of wadsleyite (Wds) along the [100] zone axis. Coherent wadsleyite–ringwoodite intergrowth, indicating the crystallographic relationships <110>_Rwd_//[010]_Wds_ and <001>_Rwd_//[001]_Wds_, was previously observed in a shocked meteorite^42^ and high-pressure experiments^43^; however, the wadsleyite SAED pattern does not match the one observed in the present study.

Euhedral and subhedral ringwoodite grains with sizes of 350 ± 200 nm occur as monomineralic aggregates in a shock vein (Supplementary Fig. S4). These grains are crystallographically randomly oriented. Coexisting phases or inclusions were not observed within the grains. The chemical formula of the grains was determined to be (Mg_1.37_Fe_0.64_Al_0.01_)Si_0.98_O_4_ using analytical TEM (Supplementary Table S1). Most of the ringwoodite grains exhibit pervasive stacking faults on {110} planes (Fig. 1). These defects have been reported in ringwoodite from many shocked ordinary chondrites^25–27^. Despite the typical microtexture, the detailed analysis of the respective grains revealed novel crystallographical features. Selected area electron diffraction (SAED) patterns of most of the grains of the ringwoodite aggregates show strong diffraction spots that are consistent with its original space group $Fd\overline{3}m$. In addition, weak diffraction spots were observed (Fig. 2 and Supplementary Fig. S5). The array of additional diffraction spots corresponds to a *d*-spacing of 0.83 nm. The {100} planes of ringwoodite and (001) plane of wadsleyite have ~0.8 nm *d*-spacings. However, diffraction spots from these planes are forbidden based on the extinction rules with respect to the electron diffraction of face-centred (ringwoodite) and body-centred (wadsleyite) cells^28^. Therefore, the SAED patterns with *d* = 0.83 nm cannot be indexed using naturally occurring olivine polymorphs, even when taking multiple diffraction into account.

**Figure 3:**
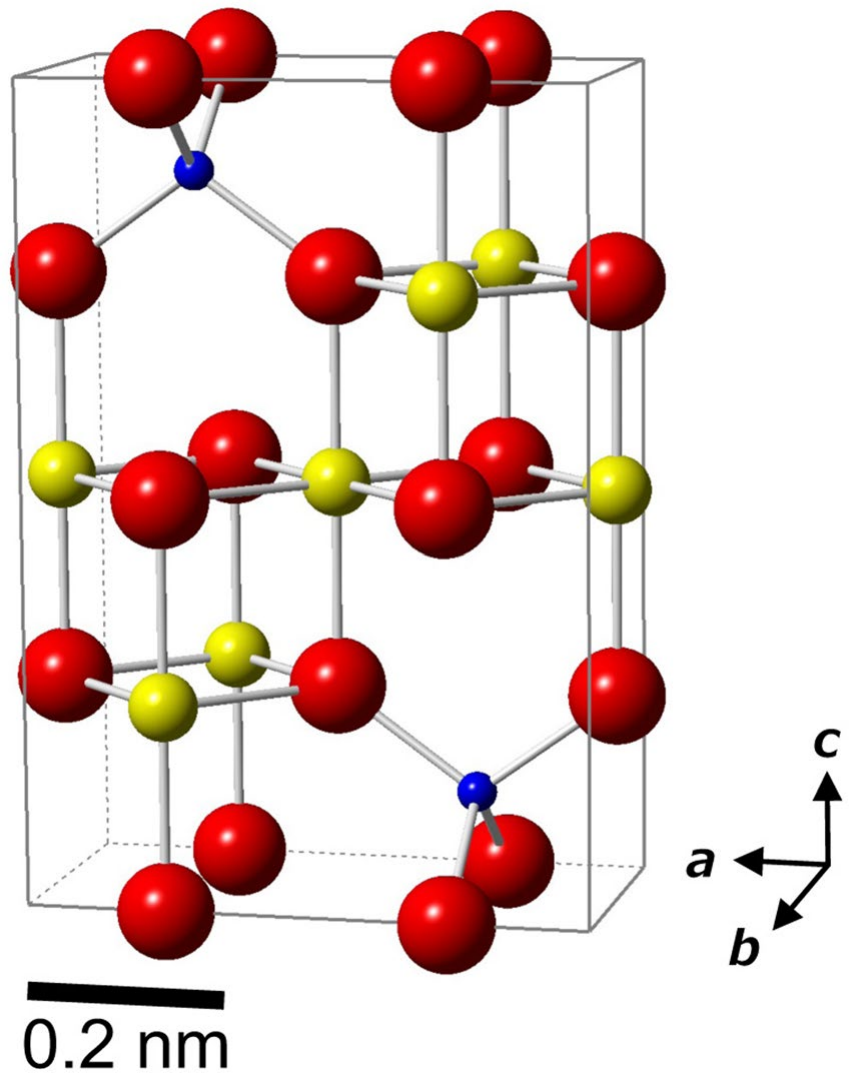
Schematic crystal structure of the newly discovered Mg_2_SiO_4_ spineloid (ε*-phase). The structure was deduced from genetic relationships between spinel and spineloid structures^21,29^. The blue, yellow, and red spheres represent Si, Mg, and O ions, respectively. The Fe and Mg ions occupy the same sites.

**Figure 4:**
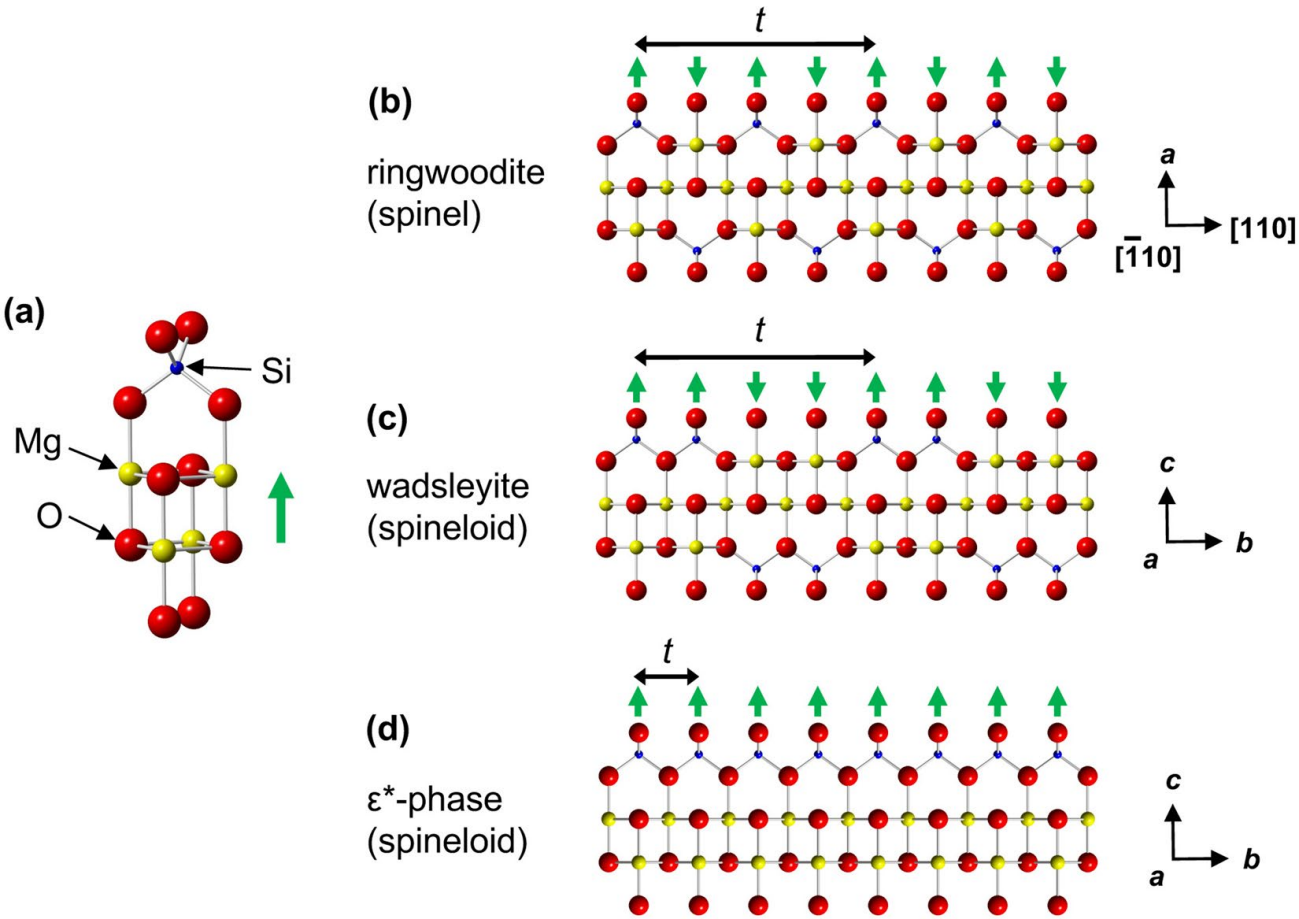
Crystal structures of Mg_2_SiO_4_-spinel and -spineloids. (**a**) Perspective view of the basic structural unit of spinel and spineloid structures. Simplified partial crystal structures of (**b**) ringwoodite and (**c**) wadsleyite based on experimental data^40,41^ and (**d**) that of the ε*-phase proposed based on hypothetical models^21,29^. The differences of these crystal structures can be characterized by periodicities of upward and downward orientations of the basic structural unit denoted by the symbols ↑ and ↓, respectively. The ringwoodite (spinel) structure has a translational periodicity (*t*) represented by …↑↓↑↓… and wadsleyite (spineloid) has a periodicity of ↓↓↑↑. The ε*-phase (spineloid) structure consists of a single orientation of the basic structural unit denoted with …↑↑↑↑…. These structures can be interchanged with each other by shear along 1/4 <$1\,\overline{1}\,2$> on {110} in the ringwoodite structure^21^.

**Figure 5:**
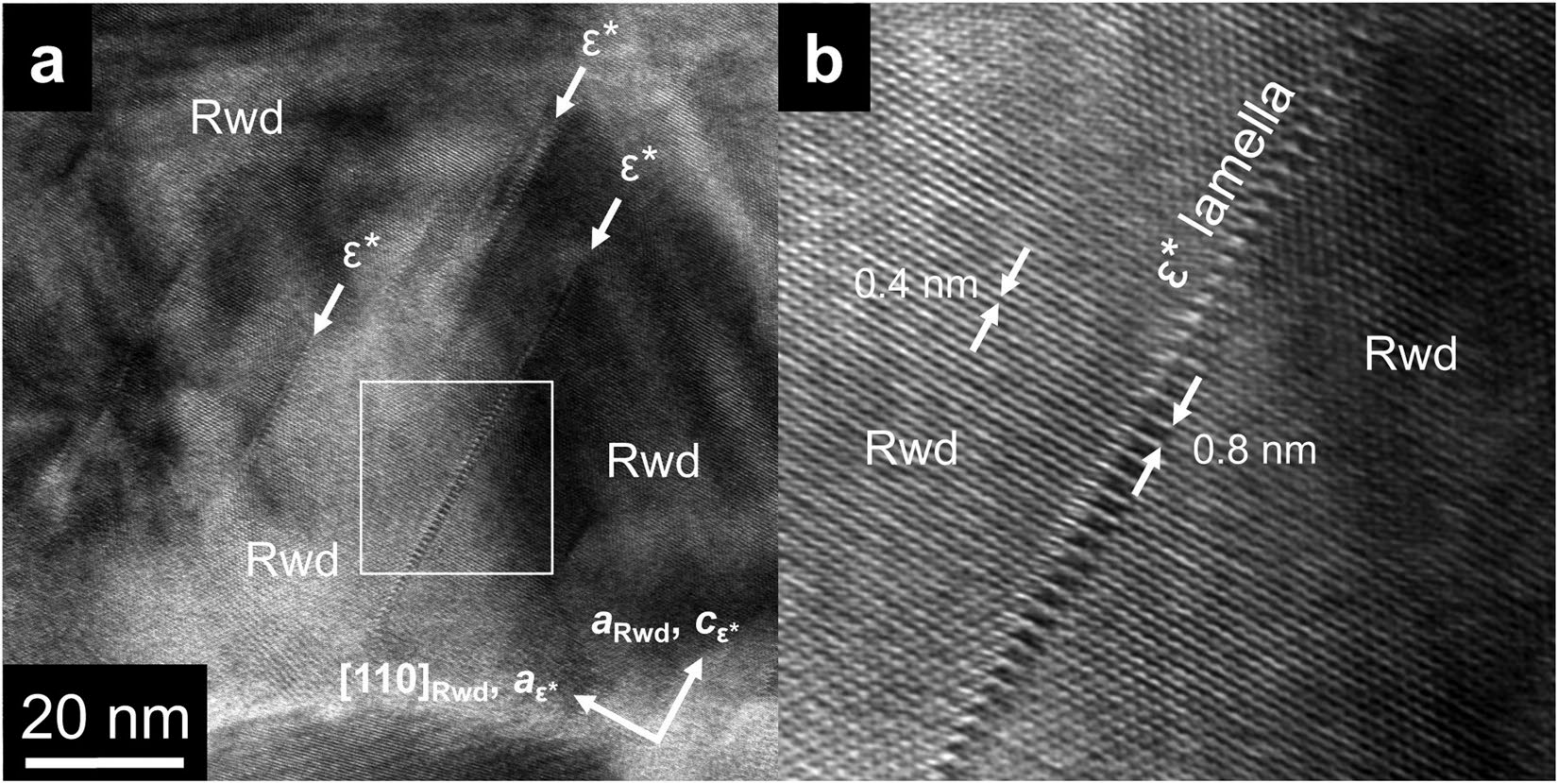
High-resolution transmission electron microscopy image of ringwoodite (Rwd) with the lamellar ε*-phase. (**a**) Thin (<3 nm) ε*-phase lamellae occurring on (110) planes of ringwoodite. (**b**) Close-up view of a ε*-phase lamella in the white box in (A). The lamella shows lattice fringes with 0.8 nm spacing of the (001) planes, characteristic for the ε*-phase. Lattice fringes with 0.4 nm spacing correspond to the (002) planes of ringwoodite.

The 0.83 nm *d*-spacing is characteristic for the unit cell of a new spineloid structure that was previously predicted as ε*-phase in Mg_2_SiO_4_ polymorphs^21^ (Fig. 3). A similar hypothetical structure was also independently predicted in the system Ni_2_SiO_4_– NiAl_2_O_4_^29^. The ε*-phase has a unit cell with nearly the same *a-* and *c*-axes dimensions and 1/4 of the *b*-axis of that of wadsleyite (Fig. 4). Weak diffraction spots overlapping with the strong SAED pattern of ringwoodite could only be indexed using the ε*-phase. The lattice parameters of the ε*-phase estimated based on the diffraction patterns are *a* = 0.578(8) nm, *b* = 0.288(3) nm, and *c* = 0.833(14) nm. The unit cell volume is 0.139(6) nm^3^. High-resolution TEM (HRTEM) showed that some of stacking faults in the ringwoodite correspond to the ε*-phase with a spacing below 10 nm (Fig. 5). In addition, the SAED patterns of ringwoodite with ε*-phase lamellae (Fig. 2 and Supplementary Fig. S5) show that both phases have a topotaxial relationship: (001)_ε_ * //{001}_Rwd_ and (100)_ε_ * //{110}_Rwd_. The crystallographic relationship can be explained in terms of periodic arrangements of a basic unit of spinel and spineloid structures (Fig. 4 and Supplementary Fig. S6).

**Figure 6:**
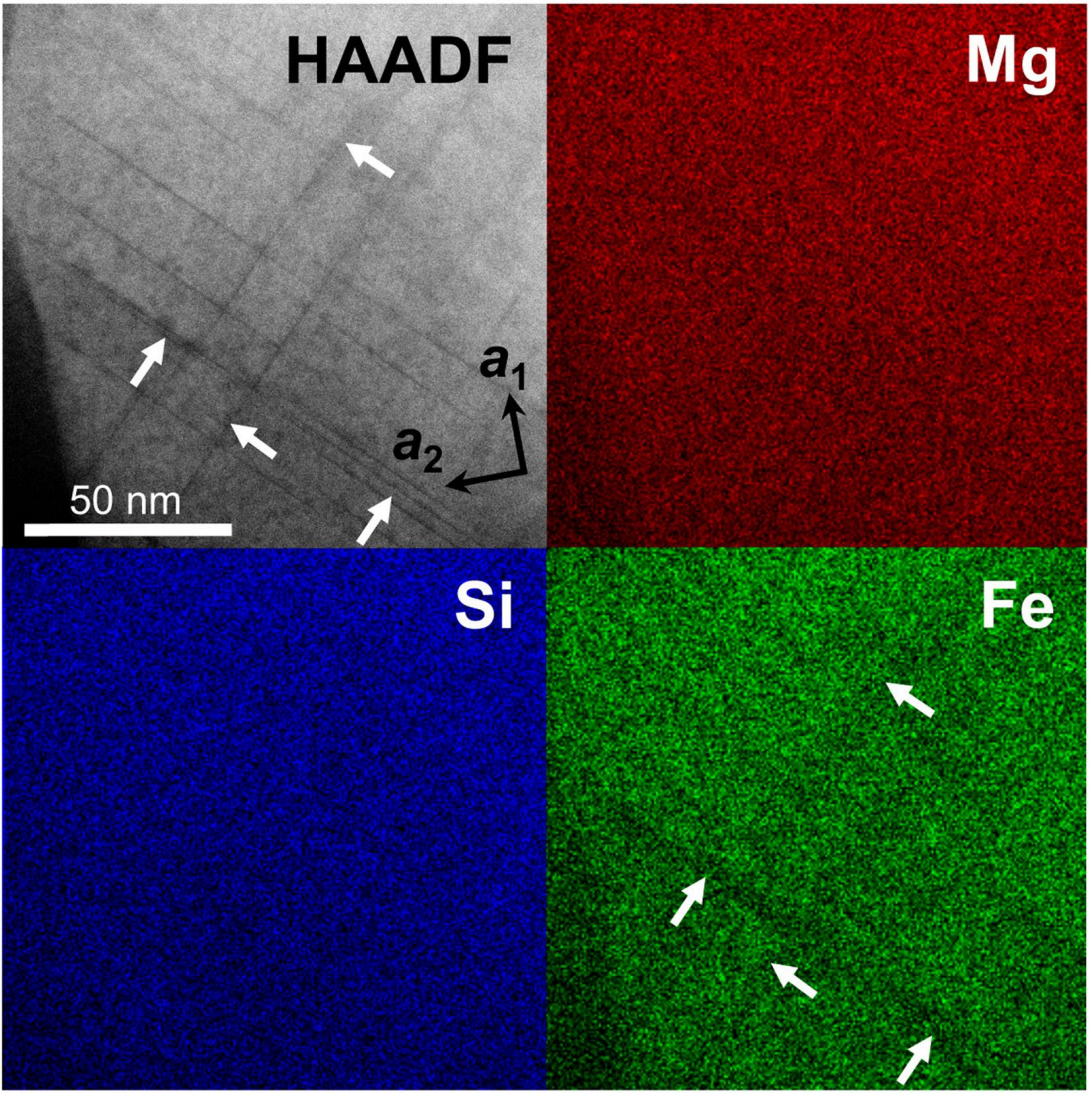
Z-contrast image and X-ray elemental maps of a ringwoodite grain with stacking faults on {101} planes. Z-contrast image obtained along the *a*-axis using the high-angle annular dark field (HAADF) technique associated with scanning transmission electron microscopy (STEM; upper left). The dark contrast in the HAADF and lower intensity in the Fe-Kα map (lower right) indicated by arrows show that the {101} stacking faults are depleted in Fe compared with the host ringwoodite.

Quantitative chemical analysis of nanometre-scale stacking faults including the ε*-phase is difficult because of sample drift during high-resolution measurements of the Ar-ion-milled sample. In addition, crystallographically equivalent stacking faults within single ringwoodite grain often overlap each other, even in an ultrathin film sample. This also complicates the chemical characterization. Nevertheless, based on Z-contrast imaging and X-ray elemental mapping using scanning transmission electron microscopy (STEM), considerable Fe-depletion within the stacking faults could be confirmed (Fig. 6).

### Formation process of the ε*-phase in a shocked chondrite

High-pressure minerals in shocked meteorites are thought to have formed by two types of mechanisms^18^. One is the solid-state transformation of host-rock minerals forming monomineralic aggregates with submicron-sized grains. The other is the crystallization of chondritic melt under high pressure forming polymineralic aggregates with larger grains (up to several µm) compared with those produced by the former mechanism. The petrographic occurrence of ringwoodite in the present study is similar to that produced by the solid-state reaction. However, its Fe concentration [Fe/(Mg + Fe) = 0.32] is significantly higher than that of olivine and ringwoodite in monomineralic aggregates in the Tenham meteorite [Fe/(Mg + Fe) = 0.25–0.26]^24,25^.

Such highly Fe-rich γ-Mg_2_SiO_4_ with a compositional range of Fe/(Mg + Fe) = 0.28–0.82 reportedly occurs in form of monomineralic aggregates in the GRV052049 L5 chondrite, although the host rock olivine is relatively homogeneous [Fe/(Mg + Fe) = 0.24–0.25], comparable with that of the Tenham chondrite^30^. The Fe-enrichment in the γ-phase is probably due to solid-state Fe diffusion from surrounding chondritic melt during shock heating. Also, olivine fragments entrapped in the shock vein of the sample investigated in the present study would have initially transformed into polycrystalline ringwoodite by a nucleation and growth mechanism without melting; they concomitantly enriched in Fe from surrounding chondritic melt during cooling at the equilibrium shock pressure (Supplementary Fig. S7). The upper pressure–temperature limit of the formation conditions of the Fe-rich ringwoodite is ~25 GPa and ~2000 °C, corresponding to the stability field of ringwoodite based on the phase diagram of the Allende chondrite^31^.

The topotaxial intergrowth between the ε*-phase and ringwoodite observed in the HRTEM and SAED patterns in the present study clearly indicates that the shear mechanism in the solid state plays the essential role in the transformation from ringwoodite to the ε*-phase. This process is achievable by shorter ion movement than that occurring during the ringwoodite–olivine transformation. In addition, atomic diffusion beyond the dimension of one unit cell is not required. The shear mechanism is therefore considered to be favourable under high differential stress or at high pressures and relatively low temperatures, where the overpressure above the olivine stability field is high but atomic diffusion is kinetically hindered. It is likely that the back-transformation of ringwoodite to olivine during shock metamorphism in the Tenham meteorite through a nucleation and growth mechanism is kinetically hindered due to the very large cooling rate of the shock veins (>10^3^ °C/s) before the release of shock pressure^32^. In such a case, ringwoodite might have metastably and coherently transformed to the ε*-phase by a shear mechanism in the stability field of olivine (Supplementary Fig. S7). As mentioned above, the ε*-phase is somewhat depleted in Fe compared with the host ringwoodite. This suggests that nanometre-scale Mg–Fe interdiffusion has occurred subsequently after the shear transformation from ringwoodite to the ε*-phase. A Mg-rich composition would be more favourable for the formation of the ε*-phase structure.

### Possible role of the ε*-phase in the deep Earth

Based on the previous topological study, the olivine ↔ ringwoodite transformation can be promoted by either a nucleation and growth mechanism or a shear mechanism^16^. In contrast, the olivine ${\rm{\leftrightarrow }}$ wadsleyite transformation can only be promoted by the former because the structurally weaker genetic relationship between olivine and wadsleyite inhibits the latter^21^. In fact, topotaxial olivine–wadsleyite intergrowth was neither observed in shocked meteorites nor in high-pressure experiments at temperatures above 1030 °C and pressures above 13.5 GPa^33^.

**Figure 7:**
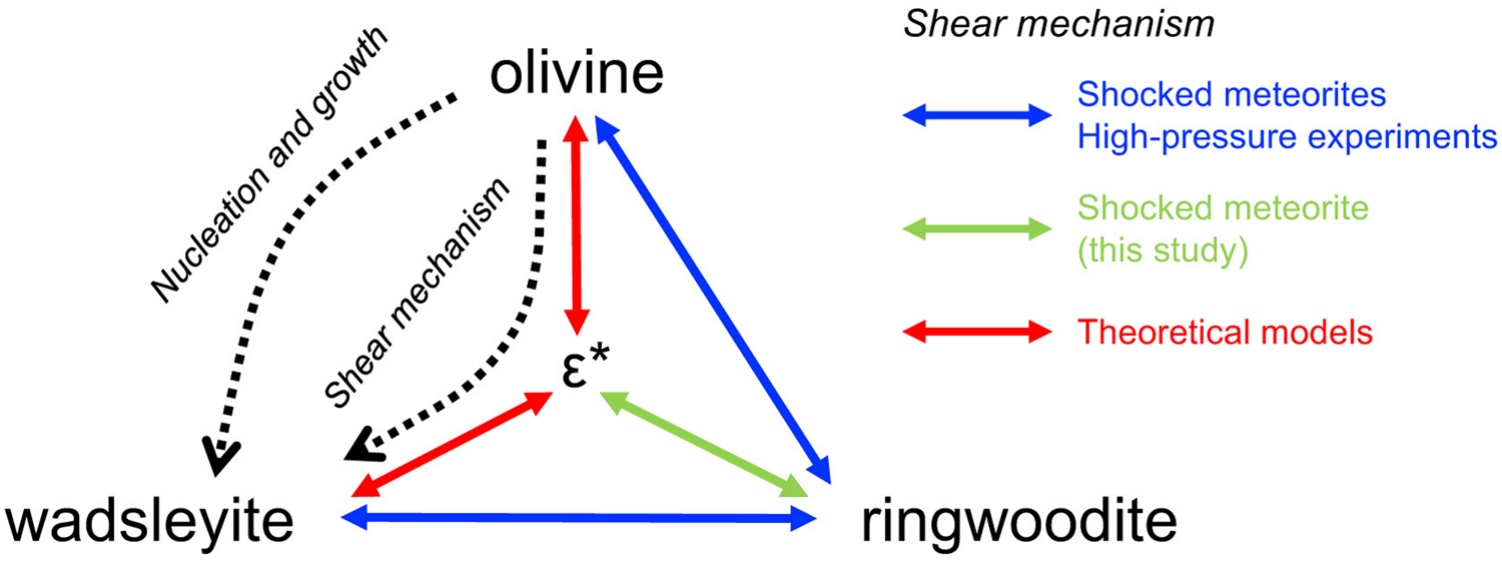
Possible high-pressure phase transformation mechanisms among the Mg_2_SiO_4_ polymorphs (modified after ref.^21^). Two-direction arrows represent the ‘diffusionless’ shear transformation mechanism between the phases. The blue, green, and red colours denote shear mechanisms previously observed in shocked meteorites^42,44^ and high-pressure experiments^5,9,43^, the present study, and theoretical models^21^, respectively. Based on the topological study of olivine polymorphs, the olivine–ringwoodite and wadsleyite–ringwoodite transformations can be intracrystalline-transformed by shear mechanisms, while the olivine–wadsleyite transformation cannot directly occur via such a mechanism but requires an intermediate ε*-phase^21^.

However, the ε*-phase may play a unique role as the relay point enabling the latter mechanism. A two-step transformation via the intermediate ε*-spineloid should facilitate the olivine–wadsleyite transformation through shearing, even if the temperature is not high enough for nucleation and growth (Fig. 7). Previous transformation experiments of pure Mg_2_SiO_4_ olivine at 15 GPa and 1000 °C demonstrated the high density of staking faults on (010) planes in wadsleyite of the run products^7,8^. Wadsleyite was assumed to have ordered oxygen sublattices but locally disordered cation sublattices. Although the disordered spineloid has not been clearly characterized, this structure could be an intermediate phase in the olivine–wadsleyite transformation^8^. Hence, we believe that some of the stacking faults of the above-mentioned sample have a structure identical to that of the ε*-phase. The ε*-phase is expected to be identified also in synthetic and natural wadsleyite in future studies.

The meteoritic ringwoodite of the present study is much more Fe-rich [Fe/(Mg + Fe) ≈ 0.3] than olivine polymorphs in the Earth’s upper mantle [Fe/(Mg + Fe) ≈ 0.1]^34^. However, the occurrence of the Fe-depleted ε*-phase in the Tenham meteorite indicates that the ε*-phase might preferentially occur in the mantle composition. The unequivocal evidence of natural ε*-Mg_2_SiO_4_ in the present study therefore highlights the geophysical importance of high-pressure phase transformations of olivine in the deep Earth. When olivine in the outermost part of the slab subducting into the MTZ is heated enough by the surrounding hotter mantle, it transforms into wadsleyite through a nucleation and growth mechanism. Meanwhile, thermal models demonstrated that the inner part of older slabs subducting fast into the upper part of the MTZ is colder than 600 °C^15^. At such low temperatures_,_ the olivine–wadsleyite transformation would be dominated by the shear mechanism via the ε*-phase, which minimizes the activation energy of the transformation. If the above-mentioned scenario is valid, the phase transformation of metastable olivine in the subducting lithosphere would be significantly enhanced (Supplementary Fig. S8).

In conclusion, the discovery of the ε*-phase in the present study indicates new pathways of diffusionless transformation mechanisms among all olivine polymorphs (Fig. 7). Further experimental and theoretical studies of the formation conditions of the new Mg_2_SiO_4_ spineloid will lead to more detailed insights into both the shock metamorphism of planetary materials and dynamics of the deep Earth.

## Methods

### Materials and analytical transmission electron microscopy

A standard petrographic thin section (32 mm × 20 mm) of the Tenham meteorite was used in the present study. The specimen used for transmission electron microscopy (TEM) was removed from the thin section. A portion of a shock-induced melt vein mounted on a single-hole molybdenum grid was processed into a thin foil by Ar-ion bombardment at 4 kV and 0.8 mA (Gatan DuoMill model 600) at Hokkaido University during previous TEM studies^35,36^ (Supplementary Fig. S3). The thin film sample was examined using a JEOL ARM-200F transmission electron microscope operated at an accelerating voltage of 200 kV at the Kochi Institute for Core Sample Research of the Japan Agency for Marine-Earth Science and Technology (JAMSTEC). We were able to identify the phases and evaluate the crystal orientations of the olivine polymorphs using selected area electron diffraction (SAED). Microtextures were determined using diffraction contrast imaging. The Z-contrast image was obtained by high-angle annular dark field scanning transmission electron microscopy (HAADF-STEM).

The chemical compositions of the samples were obtained using energy-dispersive X-ray spectroscopy (EDS) with a 100 mm^2^ silicon drift detector and JEOL Analysis Station 3.8 software. For quantitative chemical analyses, the k-factors for Na, Mg, Al, Si, Ca, Ti, and Fe were determined using San Carlos clinopyroxene standards. Theoretical k-factors were used for Cr and Mn. The intensities of the characteristic X-rays of each element were measured using a fixed acquisition time of 50 s, beam spot size of ~100 nm, and beam current of 350 pA. The thickness correction of the k-factor is described elsewhere^37^. To evaluate the reliability of the quantitative chemical analyses, San Carlos olivine (91 mol% forsterite–9 mol% fayalite)^38^ and majorite garnet of the Tenham chondrite (79 mol% enstatite–2 mol% wollastonite–19 mol% ferrosilite)^32^ were analysed with the same analytical procedures used for the ringwoodite grains containing the new silicate spineloid (ε*-phase). The Fe/(Mg + Fe) ratios obtained for olivine and garnet are consistent with literature values. X-ray elemental maps were obtained in STEM mode within an acquisition time of ~1 hour to minimize sample drift during measurements.

### Crystal structure diagnosis by single-crystal electron diffraction

In the present study, high-pressure polymorphs of olivine were identified by single-crystal electron diffraction considering *d*-spacings and interangles of reciprocal lattice vectors of respective unit cells. The reflection conditions based on the crystal symmetry of each crystal were evaluated considering the dynamical effect on electron diffraction (i.e. multiple diffraction). The following crystallographic parameters were used for the phase identification of olivine polymorphs:

Forsterite (olivine)^39^

Space group: *Pbnm* (orthorhombic)

Lattice parameters: *a* = 0.4753 nm, *b* = 1.0190 nm, *c* = 0.5978 nm

Reflection conditions: *k* = 2n for 0*kl* (*b*-glide plane; n is an integer); *h* + *l* = 2n for *h*0*l* (*n*-glide plane)

Wadsleyite (spineloid)^40^

Space group: *Imma* (orthorhombic)

Lattice parameters: *a* = 0.5698 nm, *b* = 1.1438 nm, *c* = 0.8257 nm

Reflection conditions: *h* + *k* + *l* = 2n for *hkl* (body-centred lattice); *h* = 2n for *hk*0 (*a-*glide plane)

Ringwoodite (spinel)^41^

Space group: $Fd\overline{3}m$ (cubic)

Lattice parameters: *a* = 0.8065 nm

Reflection conditions: *h* + *k*, *k* + *l*, *h* + *l = *2n for *hkl* (face-centred lattice); *k* + *l* = 4n for 0*kl* (*d*-glide plane); *h* = 4n for *h*00 (4_1_ screw axis)

ε*-phase (spineloid)^21,29^

Space group: *Pmma*

Lattice parameters: *a* = 0.5698 nm, *b* = 0.2860 nm, *c* = 0.8257 nm (estimated based on the lattice parameters of wadsleyite^40^)

Reflection conditions: *h* = 2n for *hk*0 (*a*-glide plane); *h* = 2n for *h*00 (2_1_ screw axis)

^†^All lattice parameters reflect pure Mg_2_SiO_4_.

## Electronic supplementary material

Supplementary Information
