# Supplementary material for: A new high-pressure form of Mg2SiO4 highlighting diffusionless phase transitions of olivine
Source: Sci Rep. 2017 Dec 11;7:17351. doi: 10.1038/s41598-017-17698-z (PMC5725457; doi:10.1038/s41598-017-17698-z)
Supplement: Supplementary file 1 — Supplementary Information [file 41598_2017_17698_MOESM1_ESM.pdf]

Supplementary Information

A new high-pressure form of Mg_2SiO_4 highlighting diffusionless phase transitions of olivine

Naotaka Tomioka[1,2*] & Takuo Okuchi[3]

[1]Kochi Institute for Core Sample Research, Japan Agency for Marine-Earth Science and Technology, Kochi 783-8502, Japan.

[2]Hiroshima Institute of Plate Convergence Region Research, Hiroshima University, Hiroshima 739-8526, Japan

[3]Institute for Planetary Materials, Okayama University, Tottori 682-0193, Japan

Supplementary Figure S1–S8

Supplementary References

Supplementary Table S1

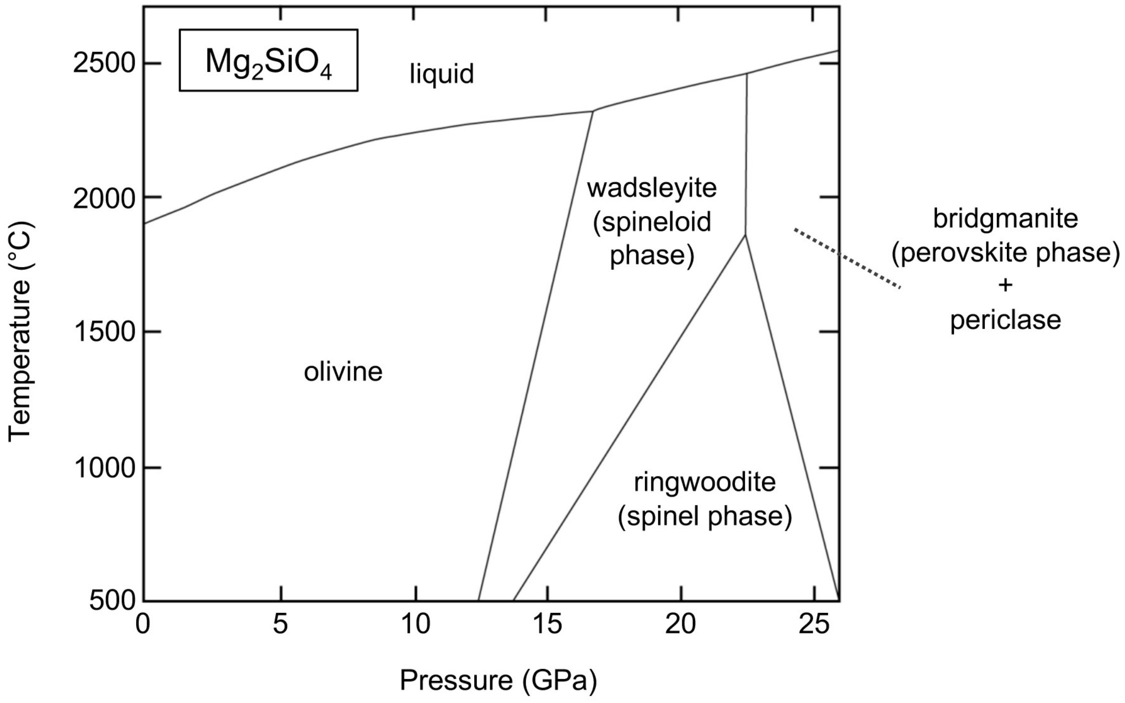

Figure S1. Phase diagram of Mg_2SiO_4 [modified after the supplementary reference 45].

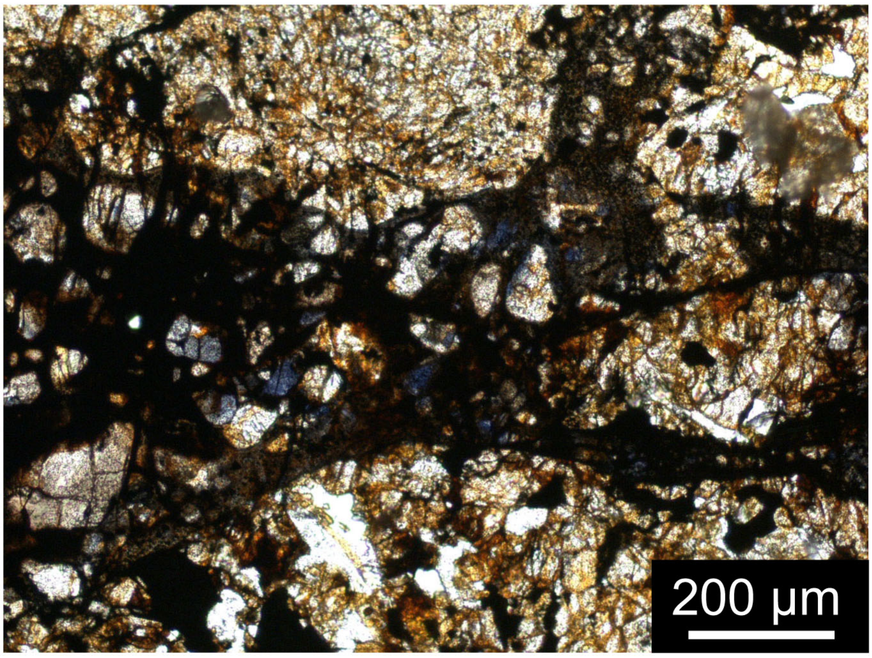

Figure S2. Optical micrograph of a typical shock-induced melt vein (shock vein) in the Tenham meteorite. Many fragments of the host-rock minerals are entrapped in the shock vein and partly or totally transformed into ringwoodite aggregates (mostly blue-colored).

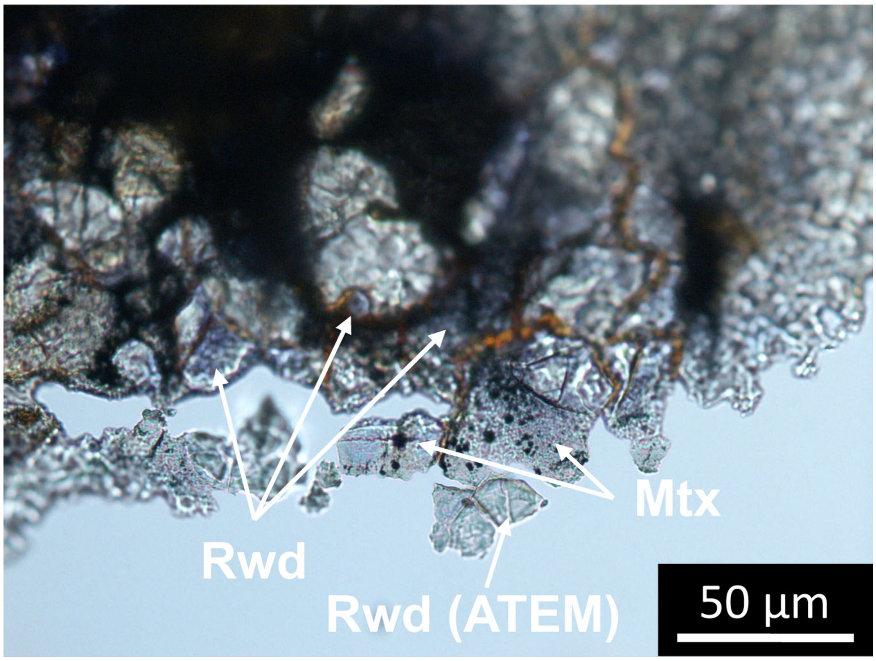

Figure S3. Optical micrograph of an Ar-ion-thinned sample from a shock vein in the Tenham meteorite. The ringwoodite aggregate (Rwd) used for analytical transmission electron microscopy (ATEM) is adjacent to the matrix of the shock-induced melt vein (Mtx) consisting mainly of majorite including Fe–S–Ni–O spherules.

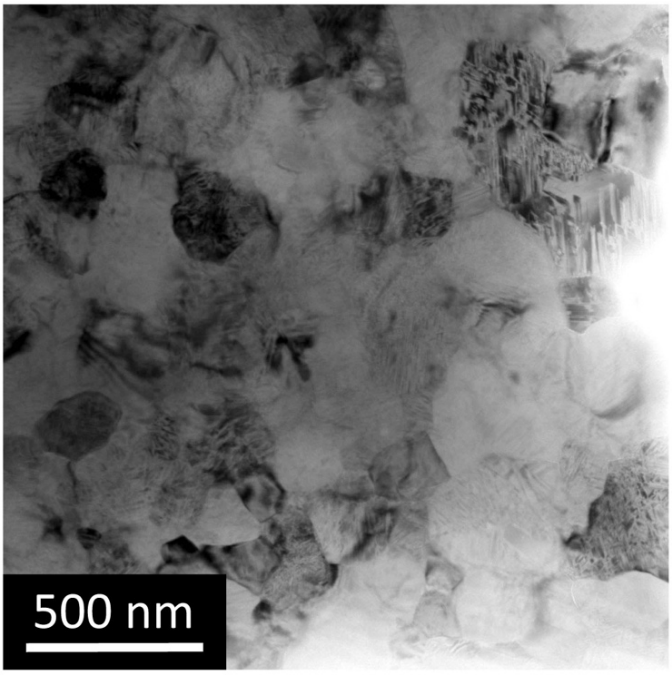

Figure S4. Transmission electron micrograph of a polycrystalline ringwoodite aggregate in the Tenham meteorite. All ringwoodite grains show frequent planar defects on crystallographic {110} planes.

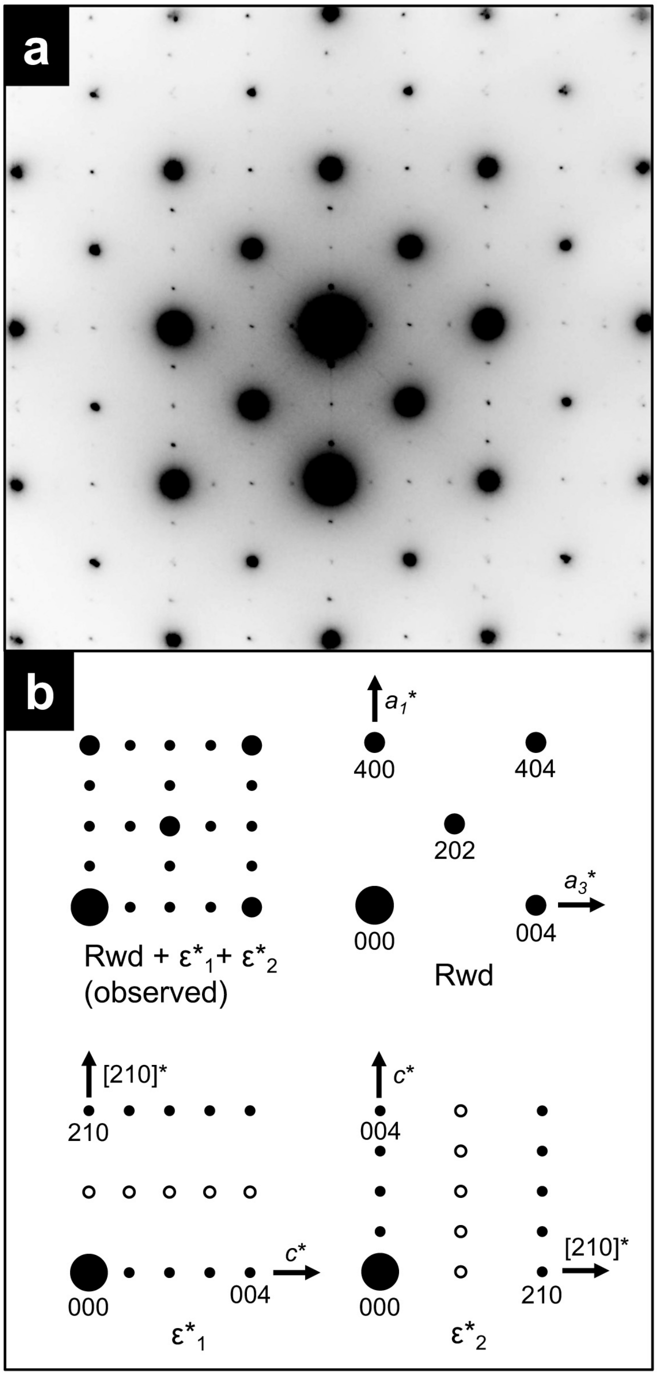

Figure S5. Selected area electron diffraction (SAED) pattern of ringwoodite (Rwd) with the ε*-phase. (**a**) SAED pattern of a ringwoodite grain along the [010] zone axis and (**b**) its schematic illustration. The grain contains the ε*-phase in two different but equivalent orientations (domain 1: ε^*_1, domain 2: ε^*_2). Domain 2 is rotated by 90° along $[010]_{Rwd}$ compared with the orientation of domain 1. The open circles denote diffraction spots from the ε*-phase caused by multiple diffraction by overlying host ringwoodite.

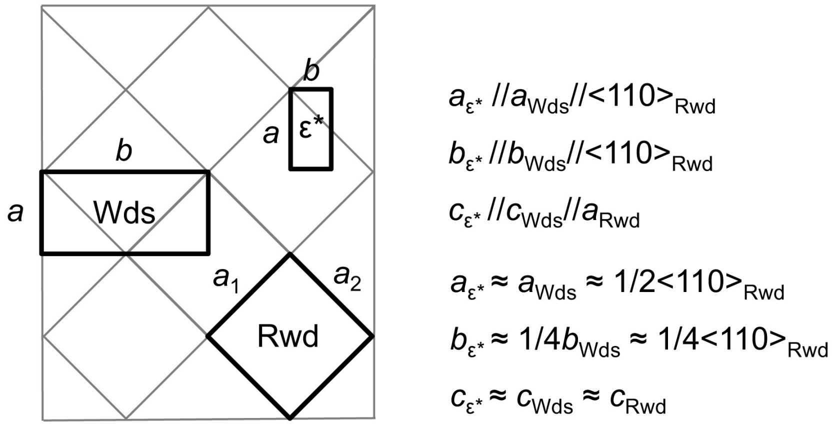

Figure S6. Axial relationships among ringwoodite (Rwd), wadsleyite (Wds), and the ε*-phase. The *c*-axes (a_3-axis for ringwoodite) of all phases are oriented perpendicular to the paper.

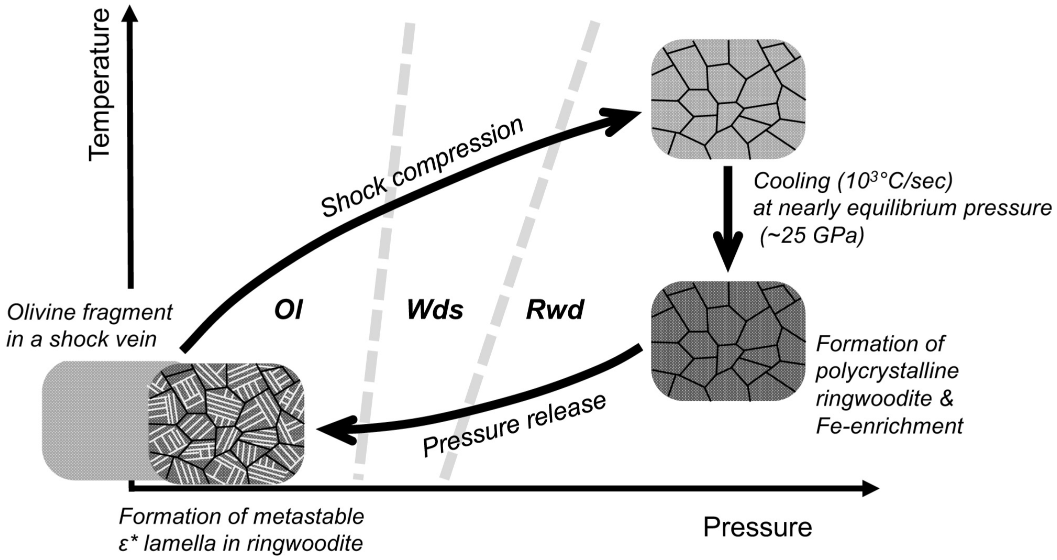

Figure S7. Schematic illustration of the formation process of the ε*-phase in shock-induced melt veins of the Tenham meteorite. The phase boundaries between Fe-bearing olivine (Ol), wadsleyite (Wds) and ringwoodite (Rwd) are simplified considering only univariant reactions. In the sample investigated in the present study, an olivine grain entrapped in the host rock was initially transformed into randomly oriented polycrystalline ringwoodite in a solid-state transformation, which concomitantly enriched in Fe from surrounding chondritic melt during rapid cooling (>10^3°C/sec)[32] at persisting equilibrium shock pressure (~25 GPa)[31]. The polycrystalline ringwoodite grains partly transformed into the lamellar ε*-phase on the {110} plane of ringwoodite by a shear mechanism during the subsequent decompression stage, at relatively low temperature at which long-range atomic diffusion is hindered.

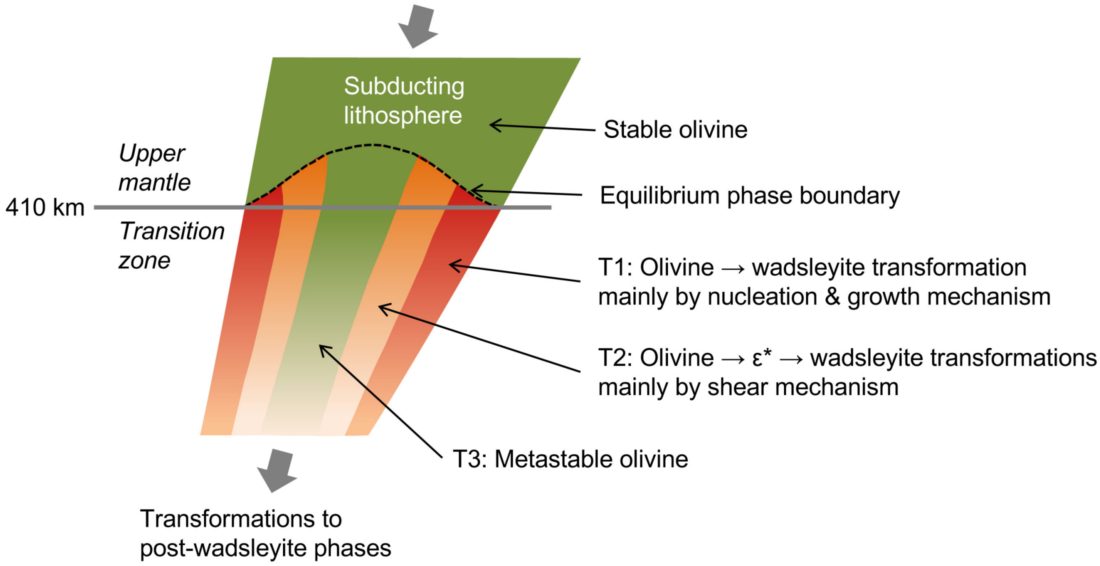

Figure S8. Conceptual diagram of olivine to wadsleyite transformation mechanisms in subducting lithosphere in the uppermost transition zone. Olivine in the outermost part of the lithosphere is heated up by the surrounding hotter mantle. When the temperature is hot enough (T_1), olivine transforms into wadsleyite through a nucleation and growth mechanism. In case the temperatures in the inner part of the slab are lower, prohibiting nucleation and growth of wadsleyite (T_2), olivine transforms into wadsleyite mainly via a shear mechanism to minimize atomic diffusion. The olivine in the much colder slab core (T_3) persists into the deeper transition zone.

Supplementary references

45. Presnall, D. C. Phase diagrams of Earth-forming minerals In *Mineral Physics & Crystallography: A Handbook of Physical Constants.* (ed Ahrens, T. J.) **2**, 248–268. (American Geophysical Union, 1995).

Table S1. Average chemical composition of ringwoodite with ε*-phase lamellae†.

Oxides	Wt.%	Standard deviation	Cation number (O = 4)
Na_2O	0.12	0.11	0.01
MgO	34.32	2.78	1.37
Al_2O_3	0.22	0.19	0.01
SiO_2	36.56	1.37	0.98
CaO	0.08	0.07	0.00
TiO_2	0.06	0.05	0.00
Cr_2O_3	0.04	0.04	0.00
MnO	0.27	0.20	0.01
FeO	28.33	3.65	0.64
Total	100*		

†Number of analyses: 20 points in 20 different grains.

‡For thin foil analyses using an energy-dispersive X-ray spectrometer equipped with a transmission electron microscope, the total weight percentage is normalized to 100%.
